# The Rooting of Stem Cuttings and the Stability of *uidA* Gene Expression in Generative and Vegetative Progeny of Transgenic Pear Rootstock in the Field

**DOI:** 10.3390/plants8080291

**Published:** 2019-08-19

**Authors:** Vadim Lebedev

**Affiliations:** Branch of the Shemyakin-Ovchinnikov Institute of Bioorganic Chemistry of the Russian Academy of Sciences, Science avenue 6, Pushchino, Moscow Region 142290, Russia; vglebedev@mail.ru; Tel.: +7-4967-33-09-66

**Keywords:** pear rootstock, adventitious rooting, stem cuttings, unintended effects, transgenic progeny, ß-glucuronidase (GUS) activity, transgene expression stability, intron-mediated enhancement, field trials

## Abstract

Adventitious rooting plays an important role in the commercial vegetative propagation of trees. Adventitious root formation is a complex biological process, but knowledge of the possible unintended effects induced by both the integration/expression of transgenes and in vitro conditions on the rooting is limited. The long-term stability of transgene expression is important both for original transformants of woody plants and its progeny. In this study, we used field-grown pear rootstock GP217 trees transformed with the reporter ß-glucuronidase (*uidA*) genes with and without intron and re-transformed with the herbicide resistance *bar* gene as model systems. We assessed the unintended effects on rooting of pear semi-hardwood cuttings and evaluated the stability of transgene expression in progeny produced by generative (seedlings) and vegetative (grafting, cutting) means up to four years. Our investigation revealed that: (1) The single and repeated transformations of clonal pear rootstocks did not result in unintended effects on adventitious root formation in cuttings; (2) stability of the transgene expression was confirmed on both generative and vegetative progeny, and no silenced transgenic plants were detected; (3) yearly variation in the gene expressions was observed and expression levels were decreased in extremely hot and dry summer; (4) the intron enhanced the expression of *uidA* gene in pear plants approximately two-fold compared to gene without intron. The current study provides useful information on transgene expression in progeny of fruit trees under natural environmental conditions.

## 1. Introduction

Pear is one of the most important fruit species in the temperate-zone countries (temperate climate zone). The world production of pear occupies the second place after apple among the deciduous fruit trees, and about 24–26 million tons of pears were produced annually in the 2010s [1]. Breeding of new genotypes of pears is carried out both by classical methods: Hybridization (in the last several centuries) or mutation techniques (at least nine mutant cultivars of pear are known [2], and by biotechnological methods: Genetic engineering since the 1990s [3], and most recently, genome editing [4]. However, whatever method is used to develop new cultivars, they need to be propagated in large quantities for commercial use. As the majority of horticultural crops, the pear is propagated by vegetative means for obtaining genetically uniform plants, whereas the sexual propagation is used by breeders. Among vegetative propagation methods, including budding, grafting, layering and cuttings, stem cutting is the most effective and economical method for producing large quantities of plants without altering the genetic constitution, especially fruit and forest trees [5,6]. For example, the reproduction of elite eucalyptus genotypes by cuttings is the main step in the creation of high-yielding, uniform, efficient ornamental nurseries and forest plantations [7]. However, the ability of cuttings and other propagules to adventitious rooting highly depends on the genotypes. Many species or even cultivars of woody plants are difficult to root, and this feature is the main obstacle to their vegetative reproduction.

Self-rooted fruit trees have a long juvenile period—for example, in an apple, tree it can take five to ten or even twelve years [8]. In order to shorten this period, trees are grown on rootstocks using grafting techniques. In addition to the acceleration of flowering, rootstocks serve to control tree size, improve biotic and abiotic stress resistance, vegetatively propagate woody species that are otherwise difficult to propagate asexually, etc. [9]. The use of transgenic rootstocks in combination with non-transgenic cultivars is promising in terms of biosafety because pollen and fruits produced by scions are non-transgenic, and, therefore, environmental and food safety risks are absent. A promising approach for improving woody fruit species, including rootstocks is the application of the more advanced biotechnological techniques, such as RNA interference, cisgenesis/intragenesis, trans-grafting, and genome editing, which are still in limited use in fruit trees [10,11]. The choice of rootstocks for pear is much more limited than it is for apple. In the world, different clones of quince are widely used as pear rootstocks, but they are not winter-hardy for the climate conditions of Russia. The genetically variable seedlings of wild pear or European pear cultivars are main rootstocks in Russia, and clonal rootstocks are used much less frequently.

Vegetative propagation of rootstocks for fruit trees is limited by their low ability to form adventitious roots. For example, it is difficult to propagate by cuttings such apple rootstocks as M9 and M26 [12] and some of the popular pear rootstocks [13]. Adventitious rooting is influenced by many exogenous and endogenous factors, including the genotype, stage of development, temperature, light, phytohormones, carbohydrates, mineral nutrients, and phenolic compounds [14]. The molecular mechanisms underlying adventitious rooting are still not completely understood [15]. The transfer of various *rol* genes from *Agrobacterium rhizogenes* were used to enhance rooting competence of rootstocks, including *rolC* to pear [16], *rolB* to apple [17], or three genes (*rolABC*) to walnut [18]. In produced transgenic plants, rooting could vary widely [19]. Formation of adventitious roots in shoot cuttings is the result of complex interactions between hormone-related pathways and a complex metabolic response [20]. Genetic transformation is a random process, and, therefore, gene transfer itself can cause the appearance of so-called unintended changes. Unintended changes may arise from different mechanisms, and there is no single direct test for its detection [21]. For this reason, unintended effects are usually evaluated by indirect signs, for example, phenotype changes. An assessment of adventitious rooting involving many biochemical, physiological, and molecular processes may be an indicator of the absence/presence of unintended changes in transgenic plants. It has been repeatedly demonstrated that the unintended pleiotropic effects of marker genes also need to be assessed, particularly in plants that are expected to remain in the field for many years and be subjected to highly variable environmental conditions [22]. Meanwhile, to the best of our knowledge, nothing is known about the impact of the transferred rooting-neutral genes on adventitious root formation in stem cuttings.

Genetic transformation and regeneration of woody plants are not routine procedures. In addition, transgene stability is particularly important for tree species that grow for many years and are subject to changes in seasons and various biotic and abiotic stresses. Stability of transgene expression under field conditions has been studied in fruit and forest trees [23,24,25], but the number of such trials with woody plants is limited, due to high research costs and regulatory restrictions for release of transgenic trees into the environment. Marker genes are often used in these studies, usually *uidA* gene, but also *gfp* gene [26]. The use of the green fluorescent protein (GFP) reporter gene is of high interest in the development of transformation protocols, since its non-destructive analysis allows to assess the progress of transgenic calli formation in the first weeks after co-culture and the combined use of GFP and kanamycin selection facilitated the early elimination of regenerating escapes [27]. Much less information is available on transgene expression in the progeny of trees, especially produced by the generative way. Evaluation of the transgene expression stability after generative and vegetative reproduction has great importance. Although the transgenic trees are propagated by vegetative means, the stability in the seed progeny is equally important, since transgenic plants can be used in classical breeding programs as donors of valuable traits. In addition, genetic engineering is specially applied to accelerate the breeding of tree species using early-flowering transgenic plants. For example, RNAi suppression of the native pear genes *PcTFL1-1* and *PcTFL1-2* resulted in early flowering phenotype that produced flowers 1–8 months after transfer to the greenhouse [28]. The authors suggest that with the use of such plants, it is possible to have four generations in nine years, whereas traditional methods require about 50 years.

In this study, we used GP217 pear rootstock of the Russian origin that has high frost resistance and is one of the few clonal pear rootstocks in Russia. Transgenic plants expressed *uidA* and *uidA*-intron marker genes [29]. In addition, plants contained the *uidA* gene were re-transformed with the *bar* gene [30]. To the best of our knowledge, these plants are the first double transformants of fruit trees (“stacked” or “pyramided” events). An assessment of unintended changes in plants that have undergone a transformation process twice is particularly interesting. The objectives of this paper were (1) to detect possible unintended effects of transgenes on the rooting ability of stem cuttings from field-grown pear rootstock trees, and (2) to evaluate the stability of transgene expression in the pear progeny obtained by vegetative (cutting, grafting) and by generative means under field conditions.

## 2. Results

The rooting frequencies of wild-type and most transgenic lines on the medium without selective agents were about 45–55% (Table 1). An increased rooting rate (66.8%) was observed in the line HB-4 with the *uidA*-intron gene, but results were not significantly different from the control plants. The line HB-1 with the *uidA*-intron gene, the line P-BK with the *bar* gene and the double transformed line T-BO demonstrated significantly lower rooting percentage compared to untransformed plants—18.8%, 17.7%, and 22.5%, respectively. Rooting of transgenic shoots on the medium supplemented with the appropriate selective antibiotic or herbicide showed that significant differences were observed between the lines carrying the *uidA*-intron gene on the medium with hygromycin (Table 1).

Our results showed that the rooting efficiency of pear semi-hardwood cuttings was influenced by the year and place. On average, the rooting percentage was higher for all genotypes in the Moscow Region (MR)—48.9% (2009) and 72.3% (2010), whereas in the Orel Region (OR) it was 27.1%, 25.8%, and 46.8% in 2010–2012, respectively (Table 2; Table 3). Most of the experiments demonstrated significant fluctuations in the rooting frequency between transgenic lines, except in 2010 (MR), when results were not significantly different—all values fit into a range of 64–85%. In general, the results of five experiments during four years revealed no clear leaders and outsiders in the rooting frequency, which would be stable during all the experiments. Only the line HB-4 can be considered as the most promising genotype, which ranked first or second in four experiments out of five, except for 2010 (MR), when a high rooting percentage was observed for all transgenic lines. The line NII-1 had a high rooting percentage in the greenhouse, but a low percentage in the tunnel.

The rooting frequency of wild-type cuttings was significantly lower compared to transgenic plants when experiments were carried out in the MR (Table 2). At the same time, the diameter of control cuttings was significantly thicker than that of transgenic ones. The diameter of cuttings did not vary significantly between genotypes when rooting was conducted in the OR (Table 3). However, in 2012, the diameter of the cuttings was the smallest for all the years of experiments. There was variability in the length of the cuttings, but the deviations for different lines did not exceed 10% of the average value.

For all rooting experiments, the average number of roots >2 mm thick per cutting was between 1.5 and 2. There was a moderate positive correlation (r = 0.66) between the number of roots and cutting diameter in 2009, when we observed a significant variation in the diameter among genotypes. In other years the variation of diameter was lower, and the correlation was weaker and did not exceed 0.48. The average length of roots >2 mm was not significantly different between the lines in the first three experiments, and, therefore, this indicator was not taken into account in 2011 and 2012.

**Figure 1:**
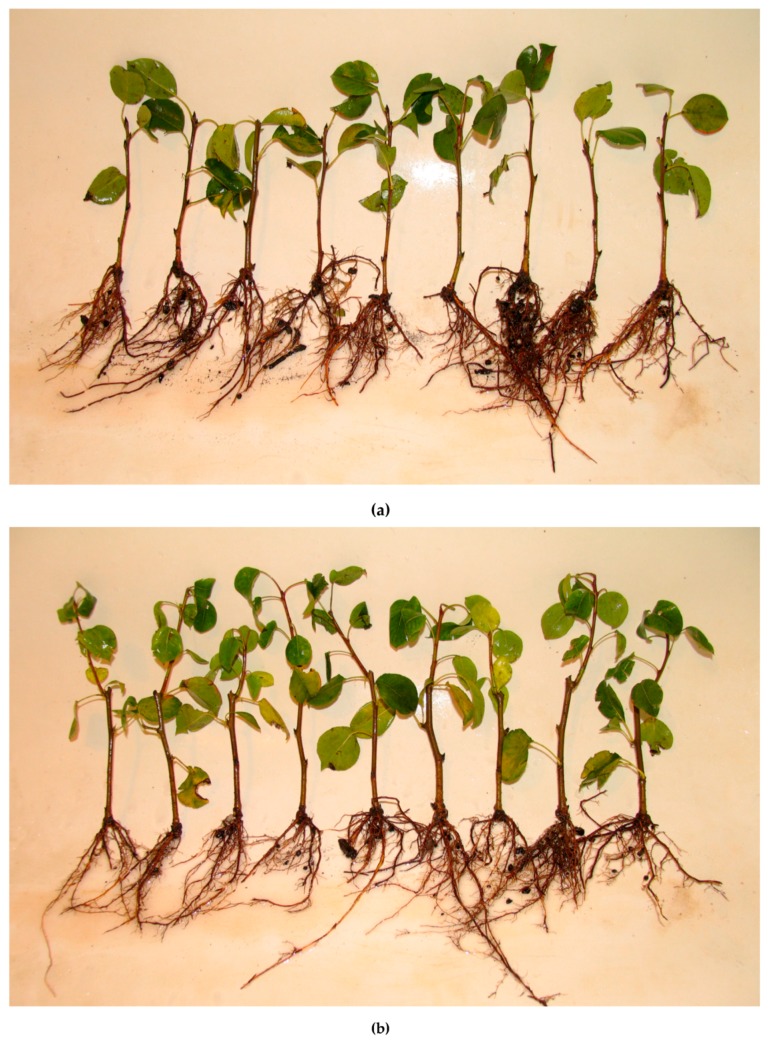
Rooted stem cuttings of pear rootstock in 2009: (**a**) Without new shoots; (**b**) with new shoots.

During experiments in 2009–2010, 39.2–47.8% of the cuttings formed new shoots from the upper lateral bud. The rate of new shoot formation sharply decreased in 2011 and 2012, down to 21.3% and 22.8%, respectively. Different genotypes showed no stability in the germination of the upper buds. For new shoot height, significant differences were observed in the OR (2010 and 2011, Table 3). The minimum length of new shoots was in 2012—38.4 mm, whereas during previous years it varied from 52 to 86 mm. Rooted cuttings with new shoots and without ones from the experiment in 2009 are presented in Figure 1. At the end of the experiment, cuttings were planted in the field for the assessment of the transgene expression level in 2010 and 2011.

**Figure 2:**
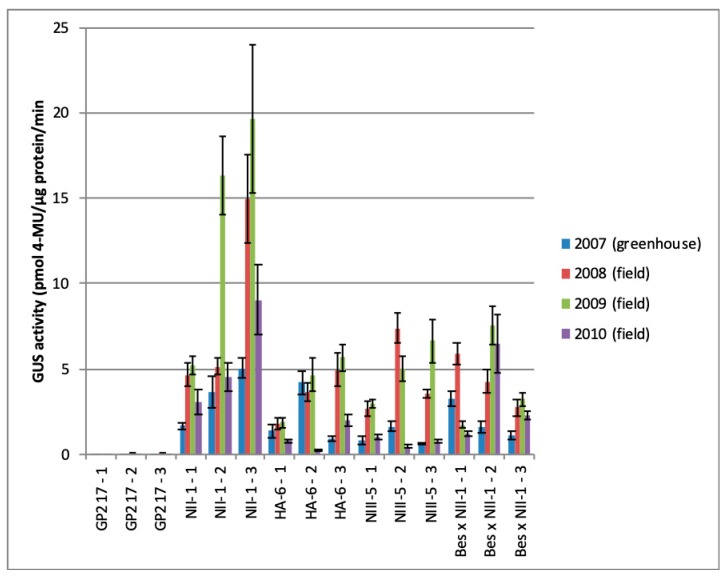
GUS activity in transgenic hybrid seedlings of pear rootstock from the harvest of 2006. Data are expressed as mean ± SD (N = 3).

The measurement of ß-glucuronidase activity level in the generative progeny of pear plants was carried out in seedlings grown from the seeds harvested in 2006–2009. These plants were tested by GUS histochemical staining before quantitative expression analysis. The GUS activity in seedlings of the 2006 harvest was measured both in the greenhouse (2007) and in the field (2008–2010) (Figure 2). Totally, eight and two fruits were harvested after pollination of Bessemyanka by pollen from the line NII-1 (*uidA* gene) and the line HA-6 (*uidA*-intron), respectively. Two fruits of the hybrid with line NII-1 and both fruits of the hybrid with line HA-6 were seedless. There were 12 standard and 16 aborted seeds in six fruits. Defective development of seeds is a specific feature of the cultivar Bessemyanka (= Seedless) is the absence of seeds, and this may be the reason for the absence of rudimentary seeds in hybrid fruits. All 12 normal seeds were planted in the greenhouse and five of them germinated. All five seedlings were tested by histochemically for the presence of the *uidA* gene and transgene was detected in three of the five seedlings.

The GUS activity depends on the year, and there was a significant drop in the expression level in 2010, possibly due to extremely hot weather conditions during the summer period (Figure 2). A significant expression decline in the year of 2010 after a relatively stable level in previous years was observed in N-2 and B-E-2 plants. Field-grown plants demonstrated increased transgene expression compared to plants in the greenhouse, up to 10 times higher for B-E-3 plant. The GUS activity in crosses between Bessemyanka flowers and transgenic pollen and in crosses between transgenic flowers and wild-type pollen was similar, but, in the first case, it was more stable over the years.

The results of the *uidA* expression analysis in seed progeny from the harvests of the years 2007–2009 are presented in Table 4.

A high variability in the transgene expression was observed in seed progeny of some lines which could reach 20 fold difference both in the greenhouse (offspring of the line NIV-2 in 2010) and in the field (offspring of the line HA-6 in 2010). As for transgenic plants from 2006 harvest, the transgene expression under field conditions was usually mainly higher than in greenhouse plants. The expression in sexual propagated transgenic plants was stable—no instances of gene silencing in three-year-old (Table 4) or four-year-old (Figure 2) plants were observed in the field. In the progeny of the wild-type GP217 rootstock and non-transgenic seedlings of transgenic lines, the 4-MU level was 0.02–0.09 pmol/µg protein/min. The expression level of the *uidA* gene with intron (average for all lines) was about two times higher than the gene without an intron both in the greenhouse (10.8 and 6.5) and in the field—9.7 and 4.9 in the year of 2010 and 43.2 and 21.8 in the year of 2011.

The expression level was also measured in the vegetative progeny of the pear obtained by budding in 2008 or grafting in 2009 (Table 5). Survival of grafted buds was on the average 76% and did not differ between wild-type and transgenic plants. Individual plants among asexual progeny of the same line demonstrated significantly more stable transgene expression compared to sexual progeny. Moreover, the expression level in cuttings was less dependent on year than in seedlings. However, the GUS activity in cuttings in 2010 was noticeably lower than in 2011, as already noted for seedlings of the harvest of 2006. The level of 4-MU in the leaves of grafted and cutting plants of pear rootstock GP217 was 0.05–0.09 pmol/µg protein/min.

## 3. Discussion

Adventitious root formation (ARF) is a complicated process involving morphological, physiological, and biological changes. This process is based on the ability of non-meristematic tissues, which do not normally form roots (e.g., stem), to dedifferentiate and develop adventitious roots [31]. Adventitious rooting is used for the reproduction of most fruit and forest tree species as one of the cheapest and simplest methods of vegetative propagation, as well as clonal micropropagation. Despite this importance, our knowledge of adventitious rooting is limited in trees [32]. Recent studies of ARF in woody plants at the level of the transcriptome and proteome [15,33,34], as well as using transgenic plants [35], have shown the participation of complex system of hormone regulation in this process, primarily auxins, which differ at different stages of development. In addition, the genes and transcription factors associated with carbohydrate and energy metabolism, wounding, and the cell cycle is also involved in the rooting process. Insertion of foreign DNA into the genome with a certain degree of probability can disrupt this process resulting in changes in adventitious rooting.

Unintended effects occur in transgenic plants for various reasons not directly related to the trait encoded by the transferred gene: Insertion and/or position effects [36]. In transgenic woody plants, a number of unintended changes are known, for example, overexpression of polygalacturonase gene in transgenic apple has led to disrupted leaf organization, silvery colored leaves, and changes in leaf abscission [37]. Some transgenic silver birch lines expressing a sugar beet chitinase IV gene were significantly more susceptible to leaf spot disease than the controls under field conditions [38]. A specific phenotype is the most frequent unintended effect conferred by a transgene in a plant [39]. Rooting ability is an important trait in woody fruit trees that are vegatatively propagated. One of the directions of genetic engineering for these plants is to improve their rooting rate. However, little is known about the change of rooting ability as an unintended effect of genetic transformation. In addition, gene transfer requires an in vitro stage (micropropagation, regeneration), which can cause such a phenomenon as a somaclonal variation. This phenomenon can be the result of individuals exhibiting various changes, including differences in the ability to organize and form organs in vitro [40]. For example, strong evidence of a somaclonal variation in the rooting ability of regenerants from leaf explants of apple has been shown [41]. It can be suggested that pyramiding genes may significantly increase the risk of deviations in double transformants.

Our studies on rooting in vitro showed that among nine transgenic pear rootstock lines transformed with the *uidA*, *uidA*-intron and *bar* genes, three genotypes demonstrated significantly lower rooting frequency compared to control (Table 1). Of these three lines, two were obtained after one transformation, and one line is the double transformant. In vitro, rooting rates are usually evaluated in transgenic plants transformed with various *rol* genes to increase rooting ability. Zhang et al. [19] reported that all three lines of the apple rootstock *Malus micromalus* with *rolC* gene significantly differed from each other by the rooting rate on the hormone-free medium. One clone of pear rootstock BP10030 (*Pyrus* type) with the *rolB* gene out of six showed a significantly reduced rooting frequency [42]. Evaluation of rooting rate in transgenic plants transformed by other genes is rarely carried out. Meanwhile, in the first report on the production of transgenic apple, it has already been shown that in vitro rooting of one of two clones was significantly reduced compared to the control [43]. On the other hand, the rooting percentage of the ‘Old Home’ pear rootstock with the GUS gene was about 95% and did not differ from the control [44]. Although the olive (*Olea europaea* L.) lines carrying the *uidA* gene and the synthetic gene for fungal resistance did not show a significant difference on rooting frequency compared to the control, it was found that the number of roots per explant and length of the main root were significantly different between transgenic and wild-type plants [45].

It is known that unintended effects could have both positive and negative effects on the resistance of transgenic trees. Gypsy moth larvae (*Lymantria dispar* L.) showed a significant reduction in survival on one transgenic aspen (*Populus tremuloides* Michaux) line with reduced lignin relative to all three other lines [46]. On the other hand, there was a significant increase in leaf damage by slugs on Bt aspen (*P. tremula* × *P. tremuloides*) compared to wild-type leaves [47]. In both cases, the reason was the change in the phytochemical profiles of leaves caused by the random insertion of genes. Such insertions can also affect the complex process of adventitious rooting. Improved rooting of stem cuttings is a useful trait, and on the contrary, a poor rooting ability may reduce the commercial value of the transgenic genotype, especially if it is a rootstock propagated by cuttings and not a cultivar propagated by grafting. In order to detect possible changes, we carried out adventitious rooting of stem cuttings from field-grown trees for several years.

The rooting of pear rootstock cuttings was influenced by various factors. A higher rooting frequency in the MR (Table 2) may be associated with both rooting conditions and the date of cutting collection. Rooting in the MR was carried out in the greenhouse where conditions were possibly more favorable than in a film tunnel in the OR. In addition, the interval between the dates of cutting collection in the MR and OR was about two weeks. The year of the experiment also had a pronounced effect on rooting of cuttings. The best season for rooting was 2010 for the MR, when all factors were optimal, and there were no significant differences between the transgenic lines. It is assumed that under optimal conditions (in the year of 2010—the MR) the rooting potential was fully realized and all lines demonstrated similar rooting rate. For the OR, the best season was 2012 (Table 3). This indicates that rooting success depends on many factors.

In addition to genetics, the rooting of cuttings can be influenced by the physiological and biochemical status of the mother plants. Growing conditions (light, temperature, availability of water and nutrients) affect the content of endogenous auxin, carbohydrates, mineral nutrients, phenolic compounds, and thus, rooting [14]. A deficiency of any important element, such as carbon or macronutrients, can limit the adventitious rooting either at the system level, interfering with the main physiological processes, or by local influence on the processes associated with induction, initiation and expression of adventitious roots [20]. The temperature to which stock plants are exposed can potentially affect the quality, quantity, and rootability of the cuttings they produce [48]. The 2010 extraordinary summer heat in western Russia [49] could affect the state of the original plants. It is unlikely that the high temperature affected the cuttings themselves, as they were cut before the onset of the hot period (in the OR). Most likely, there were high temperature effects on the rooting process in the OR and growth of pear trees during summer 2010, which affected the cuttings collected in the next 2011.

The length and diameter of cuttings can affect the rooting efficiency. The average length of the cuttings in all experiments varied slightly from 123 to 134 mm, except for 2010 (MR), when it was 103 mm. Such small variations in length have practically no effect on rooting. For example, cuttings of *Eucalyptus grandis* × *E. urophylla* of 8, 10 and 13 cm did not differ by rooting rate [50]. OuYang et al. [51] reported that the optimal cutting length for better rooting of Norway spruce [*Picea abies* (L.) Karst.] was 9–12 cm. A significant decrease in the rooting frequency of non-transgenic control in the MR may be due to the increased diameter of the cuttings. This characteristic is an important factor as it may be associated with the endogenous auxin and carbohydrate contents of the tissues [52]. Exadaktylou et al. [53] investigated the rooting of hardwood cuttings of the ‘Gisela 5′ cherry rootstock and found that cuttings with a diameter of 12–14 mm did not form roots, as opposed to diameter of 6–8 and 9–11 mm, and callus formation was maximum in the thinnest cuttings. Our experiment in 2009 demonstrated that cuttings of the same length that were greater or smaller in diameter compared to a certain diameter (too thick as GP217 or too thin as P-BU and T-BO) had lower rooting rates. Apparently, the thinnest pear cuttings were nutrient deficient, whereas in the thickest cuttings juvenility could be reduced. Higher rooting efficiency for Norway spruce was obtained by cuttings of 0.3–0.4 cm diameter [51].

The correlation between the cutting diameter and the number of roots >2 mm was found in 2009, when the thickness of cuttings was most variable (Table 2). Thus, the nutrient content of the cutting is important for root growth. The correlation was weaker if the diameter varied within 1 mm as in the years of 2010–2012. The average length of roots >2 mm depended neither on the rooting frequency, nor on the size of cuttings. The minimal percentage of pear cuttings formed new shoots during rooting was observed in 2011 and 2012. The reason may be that the hot summer in 2010 weakened pear trees that produced poor quality shoots next year (2011) and thin cuttings which have low nutrient reserves (2012). The minimal length of new shoots for all our experiments in 2012 confirms our last supposition. The effect of genotype both on the length of roots >2 mm and height of new shoots was negligible.

In sum, the single and repeated transformations of pear rootstocks apparently did not lead to the unintended effects on rooting ability, as we did not find transgenic lines demonstrating stable enhancement or reduction of rooting rate. Two original (HB-1 and T-BO lines) and one double (the P-BK line) transformants showed a significant decrease in rooting of microshoots in vitro (Table 1), but this feature was not confirmed for rooting stem cuttings during several years under open-air conditions (Table 2 and Table 3). It is possible, however, that the line HB-4 has an increased ability to adventitious rooting under non-optimal conditions. To the best of our knowledge, the assessment of the rooting of stem cuttings from woody plants transformed with neutral to rooting genes was not described earlier. For improving the rooting ability of rootstocks different genes from *A. rhizogenes* were used, and results of these studies are inconsistent. Transgenic pear rootstock lines with the *rolB* gene rooted much better than control and did not differ between themselves [42]. However, significant differences in rooting of hardwood cuttings were found among individual transgenic clones of *Populus* × *canescens* × *P. grandidentata* expressing *rolB* gene, with a range of 12.5–88.9% for the gene under 35S promoter, and a range of 20–100% for the gene under heat shock promoter [5]. Cuttings collected from two clones of M9 apple rootstock trees carrying *rolB* gene significantly differed in their rooting rate (67% and 43%) [54]. At last, only one clone of three riT-DNA Colt cherry rootstock clones rooted at the wild type level and two transgenic clones did not root at all [55]. Moreover, Fladung et al. [56] observed the formation of root suckers only in aspen trees with rbcS-*rolC* gene in the field, but not in plants with 35S-*rolC* gene, although shoot formation from roots was easy induced under in vitro conditions. Possibly, the constitutive expression of *rolC* gene induces pleiotropic changes in the whole plant, including the root system as a consequence of the altered phytohormonal balance.

Double transformants did not show clear and stable differences in phenotype and rooting ability compared to the primary transgenic lines or wild-type plants. As far as we know, similar studies with double transformed woody plants have not been conducted previously, but unintended changes were found in stacked transgenic annual species produced by re-transformation [57] or conventional breeding [58]. We found no relationship between the rooting ability of in vitro shoots and stem cuttings of transgenic pear genotypes. Depending on the year, the rooting rate of transgenic line could be either higher or lower compared to values under in vitro conditions. As mentioned above, the reduced rooting frequency of three transgenic lines *in vitro* (Table 1) was not confirmed by decreased rooting of stem cuttings. However, the line HB-4 demonstrated maximal rooting *in vitro* and high rooting capacity of semi-hardwood cuttings. Smolka et al. [54] reported that the rooting rate of cuttings of apple rootstock with *rolB* gene was lower than *in vitro* shoots, possibly due to weak growth in the field.

Our study has shown the stability of the foreign gene expression in the generative progeny as gene silencing was not observed in field-grown pear seedlings over three or four years (Figure 2, Table 4). However, expression of the ß-glucuronidase gene could vary greatly over the years, as previously reported by Maghuly et al. [59] on *Prunus subhirtella* plants in the screenhouse. We did not observe a significant decrease in the expression level in the transgenic seed progeny after transfer from the greenhouse to the field, which can be caused by stress-induced by external conditions changes. On the contrary, some plants demonstrated increased expression under field conditions. Thus, our results do not agree with data obtained by Maghuly et al. [59], which showed that the GUS activity in *Prunus subhirtella* lines in the greenhouse varied from approximately equal to about 10 times higher than in the screenhouse. However, stress did affect the transgene expression level: A decrease in the GUS activity was observed in most plants in the extremely hot and dry summer of 2010 (Figure 2). Such changes in transgene expression levels under the influence of environmental conditions in the field are often accompanied by changes in DNA methylation levels [23]. An indicator of high-temperature stress is a significant (2.5–3 times) increase in the level of polyphenols and flavanes in both wild type and transgenic pear fruits in 2010 [60]. It should be noted that in crosses with the cultivar Bessemyanka, the decrease in *uidA* gene expression was observed to a lesser extent. Perhaps this is due to the fact that the Bessemyanka is an old traditional cultivar that is well adapted to local conditions. The GUS activity levels in progeny from male and female transgenic parents were similar. The maximal level of *uidA* gene expression was observed in 2011 (Table 4), apparently due to favorable weather conditions. The transgenic pear seedlings did not display obvious phenotype changes.

The majority of experiments on intron-mediated expression (IME) was performed using *uidA* as a reporter gene [61]. The presence of a large number of individual plants expressing the *uidA* gene with and without an intron (Table 4) allowed us to evaluate the effect of the intron on expression. The intron increased the GUS activity in pear plants, on average, by 1.7 times in the greenhouse and two-fold in the field. These results in pear sexual progeny are quite consistent with our previous data, where the enhanced expression in the original pear transformants by 2.7–3.5 times in the greenhouse in 1998–1999 [29] and by 1.9–2.4 times in the field in 2001–2005 was reported [24]. This enhancement rate is typical for dicots, in which it usually is in the approximately 2- to 10-fold range, whereas it can be more than 100-fold in monocots [62]. Although IME has been known on plants for more than 30 years, the mechanism of IME is largely unknown, due to the complexity of this phenomenon both on the regulatory level and on the level of its action [61]. Studies on IME are conducted mainly on model plants [63] or monocots [64], but there are almost no reports on trees.

We also evaluated the expression stability in vegetative progeny obtained by grafting or cutting (Table 5). Grafting of transgenic scions on wild type rootstocks did not change the transgenic phenotype encoded by foreign genes, including dwarfism [16] and early flowering [28] in pear plants, and suppression of polyphenol oxidase gene in apple [65], that was later commercialized as nonbrowning Arctic^®^ apples. Resistance to glyphosate in transgenic *P. trichocarpa* × *P. deltoides* lines was maintained after propagation by cuttings [66], but quantification of the expression level in vegetative progeny was not reported in these studies. The foreign gene expression in pear plants produced by vegetative means was stable, and gene silencing was absent. In addition, expression variability in vegetative progeny was significantly lower compared to seedlings both among individual plants and years. Freiman et al. [28] reported that out of five transgenic pear seedlings obtained from a plant with RNAi suppressed *MdTFL1* gene, two flowered in 2.5 and 7 months, and the rest did not bloom after 36–45 months. This may serve as an indirect confirmation of a large variation in the blocking expression in seed progeny. On the other hand, seedlings of apple with RNAi suppressed the *MdTFL1-1* gene [67] or overexpressed the *BpMADS4* gene [68] bloomed prior to 40 weeks after seed sowing. The survival rate of grafted transgenic buds did not differ from the wild type ones. Smolka et al. [54] showed that the *rolB* gene did not have a negative effect on the budding of the apple rootstock. As for the seedlings from the harvest of 2006, the GUS activity in cutting-derived plants was lower in the year of 2010, apparently due to less favorable weather conditions.

## 4. Materials and Methods 

### 4.1. Plant Material

Two groups of transgenic pear rootstock GP217 (*Pyrus communis* L.) plants and untransformed control were used in this study. The first group of transgenic plants was generated by *Agrobacterium*-mediated transformation of leaf explants with the binary plasmid pBI121 (35S-*uidA* and nos-*nptII* genes) or p35SGUSint (35S-*uidA* with intron IV2 of the potato *ST-LS1* gene and nos-*hpt* gene) as described by Lebedev and Dolgov [29]. The upper leaves of *in vitro* rooted shoots were inoculated with agrobacterial suspension for 40–50 min and co-cultivated in the dark on regeneration medium without antibiotics for 3–4 days. For shoot regeneration explants cultured on media with 500 mg/L cefotaxim and 25 mg/L kanamycin or 5 mg/L hygromycin. The plasmid pBIBar containing the 35S-*bar* and the nos-*nptII* genes was used to produce the second group of plants. Both wild-type plants and transgenic lines expressing *uidA* and *nptII* genes were used for the transfer of the herbicide resistance gene as described by Lebedev et al. [30]. The original transformants were selected on medium with 25 mg/L kanamycin, whereas double transformants were selected on medium with 5 mg/L phosphinotricin. In order to evaluate the rooting ability of pear rootstock, non-transgenic GP217, transgenic GP217-*uidA*-intron (line HB-1 and HB-4), transgenic GP217-*uidA* (line NII-1 and NIII-2), transgenic GP217-*bar* (line P-BK and P-BU), and re-transformants (line T-BO, T2-BF, and T2-ES) were chosen. 

### 4.2. In Vitro Rooting

The shoot tips were cut from the proliferating cultures and transferred to 250 mL glass jars containing 50 mL of nutrient medium containing Quoirin and Lepoivre [69] mineral salts with a half content of NH_4_NO_3_, MS [70] vitamins, 0.5 mg/L IBA, 10 g/L sucrose and 7 g/L agar. Two series of experiments were conducted: The first did not contain any selective agents, the second included 15 mg/L kanamycin, 3 mg/L hygromycin, or 5 mg/L phosphinotricin. All media were adjusted with 1N KOH to pH 5.6–5.8 and autoclaved for 20 min at 121 °C and 1 atm. The vitamins, IBA and selective agents were filter-sterilized (Millipore, 0.22 µm) and added to the rooting medium after autoclaving. In each treatment, there were three replicates of 8, 10, or 12 shoots per jar for each genotype. The experiments were repeated 4–5 times. The shoots were grown at 23–25 °C with a 16 h photoperiod. Rooting percentage was recorded after six weeks. There was approximately a one-year interval between the first and second transgenic group rooting *in vitro*.

### 4.3. Cutting Experiments

The field site was located in the Orel Region of Russia (OR) and transgenic pear rootstock lines carrying reporter and herbicide resistance genes were planted in accordance with the permission of the Russian Inter-Agency Committee on Genetic Engineering Activity in 2000 and 2001, respectively [24]. Rooting of semi-hardwood cuttings was tested in the Moscow Region (MR) during two consecutive seasons (2009–2010) and in the OR during three consecutive seasons (2010–2012). Actively growing shoots (70–90 cm in length) were excised from the canopy of donor self-rooted non-transgenic and transgenic trees in late June (for the OR) or early July (for the MR). For transportation to the MR (about 270 km) the shoots were wrapped in wet paper to prevent desiccation. The cuttings (10–15 cm in length) were cut immediately before the auxin treatment, and the top 2–3 leaves were kept. The basal end of each cutting was dipped to a depth of 3–4 cm in IBA solution (50 mg/L) for 12 h before inserting into the rooting substrate. The rooting substrate was a mixture of peat and sand (1:1) of depth 15–20 cm, under which was a layer of drainage (coarse sand). Length and base diameter of each cutting were measured before planting. The cuttings were incubated in the greenhouse (MR) or polyethylene tunnel (the OR) under natural light conditions. The cuttings were kept under intermittent mist (10–15 s every 5–15 min depending on temperature conditions) during daylight hours, maintained at 70–90% relative air humidity. About 100–150 (in 2009) or about 100 (in 2010) cuttings were planted in the MR and 200 (in 2010) or 100 (2011, 2012) ones in the OR for each genotype. The experiments were designed as a completely randomized block in four replicates. The data were recorded after three months. The measured parameters included the number of rooted cuttings, the number of roots >2 mm thick and their length per cutting, the number of cuttings with new shoots and their height. 

### 4.4. Obtaining Generative and Vegetative Progeny of Pear

The flowers of untransformed plants and transgenic lines of pear rootstock GP217 were used for pollination in 2006–2009. Pollen was collected from field-grown plants of different pear cultivars. Several tens of flowers for each transgenic line and wild-type plants were pollinated with non-transgenic pollen every year, and several tens of hybrid seeds were sown. The old local pear cultivar Bessemyanka was pollinated with two transgenic lines carrying *uidA* gene (line NII-1) and *uidA*-intron gene (line HA-6) in 2006. Seeds were removed from the fruits, stored at 4 °C for three months to break dormancy and were germinated in the greenhouse in the following spring. 15–20 seedlings were grown for 1–2 months in pots and evaluated by histochemical GUS assay. Three or four individuals were randomly selected from GUS-positive plants for each line and grown in the field up to four years. In total, almost 100 transgenic pear hybrids were studied. Wild-type pear rootstock and four transgenic lines with reporter genes (HB-1, HB-4, NII-1, and NIII-2) were vegetative propagated by grafting on field-grown pear seedlings in the summer of 2008. The ten plants were grafted for each genotype using T-budding method. 

### 4.5. GUS Histochemical and Fluorometric Assays

The progenies resulting from the crosses with transgenic pear plants were screened for GUS expression by a qualitative histochemical GUS assay [71]. Young leaves from seedlings were tested using X-Gluc as substrate. Three (2007, 2009, 2010) of four (2008) plants were randomly selected from GUS-positive progeny of each transgenic line for quantitative assessment of GUS activity. Three leaves from the middle of the shoot were used in one-year-old greenhouse-grown plants (early June) and a mixture of several leaves from the three top lateral shoots in field-grown plants (early July). Fluorometric assay was conducted as described by Scott et al. [71] using 4-methyl-umbelliferyl-β-D-glucuronide (MUG) as substrate. Fluorescence of released 4-methylumbelliferone (4-MU) was measured using the Infinite 200 multifunctional microplate reader (Tecan Group Ltd., Switzerland) with excitation at 360 nm and emission at 465 nm (Pushchino Center for Collective Use of Science Equipment). The amount of total soluble protein was determined according to the Bradford method with bovine serum albumin as standard [72]. GUS activity was expressed as picomoles of 4-MU produced per minute per milligram of protein. Pear crosses from 2006 were evaluated during four years in the greenhouse (2007) and in the field (2008–2010). Other seed progenies were evaluated once as greenhouse-grown one-year plants (2010) or three-year plants in the field (2010 and 2011). The grafted plants were tested for GUS activity in 2009 and rooted cuttings in 2010–2011 in three randomly selected individuals under field conditions. 

### 4.6. Statistical Analysis

Data were analyzed by analysis of variance (ANOVA) and Duncan’s multiple range test at a significance level of 0.05 using the Statistica software (Statsoft Inc., Tulsa, OK, USA).

## 5. Conclusions

In summary, the single and repeated transformations of clonal pear rootstocks by marker and herbicide resistance genes did not result in the unintended effects on adventitious root formation in semi-hardwood cuttings from field-grown trees. We demonstrate the stable expression of reporter ß-glucuronidase genes and absence of gene silencing in the generative and vegetative progeny of pear up to four years under field conditions. Year-dependent variation in the foreign gene expression was observed, and expression levels were decreased in extremely hot and dry summer. The intron in the *uidA* gene increased the GUS activity in pear plants approximately two-fold compared to the no-intron construction. The current study provides useful information on transgene expression in progeny of fruit trees under natural environmental conditions.

## Figures and Tables

**Table 1:** Rooting pear rootstock in vitro.

Genotype	Gene	Selective Agent	Rooting Rate, %	
			sel. Agent −	sel. Agent +
GP217	-	-	47.2 ab	-
HB-1	*uidA*-intron	Hy	18.8 c	26.0 b
HB-4	*uidA*-intron	Hy	66.8 a	52.9 a
NII-1	*uidA*	Km	54.8 ab	48.3
NIII-2	*uidA*	Km	43.3 b	36.7
GP217	-	-	54.0 a	-
P-BK	*bar*	PPT	17.7 b	19.9
P-BU	*bar*	PPT	36.5 ab	33.3
T-BO	*uidA* + *bar*	PPT	22.5 b	23.1
T2-BF	*uidA* + *bar*	PPT	50.9 a	37.5
T2-ES	*uidA* + *bar*	PPT	44.8 a	31.9

Different letters indicate significant differences between treatments (*p* < 0.05). Hy—hygromycin, Km—kanamycin, PPT—phosphinotricin.

**Table 2:** Rooting of pear rootstock stem cuttings in the Moscow Region.

Genotype	Cutting Length, mm	Cutting Base Diameter, mm	Rooting, %	Number of Roots >2 mm per Cutting	Mean Length of Roots >2 mm, mm	Cuttings with New Shoots, %	New Shoot Height, mm
				2009			
GP217	132.4 a	5.3 a	29.4 c	2.0 ab	116.5	42.5 b	45.3
HB-1	123.0 ab	4.7 b	59.9 ab	1.8 bc	134.5	49.5 ab	67.5
HB-4	135.6 a	4.7 b	71.6 a	2.2 a	109.4	39.8 b	56.2
NII-1	121.0 ab	4.4 b	63.5 ab	1.5 cd	97.5	51.5 ab	54.7
NIII-2	125.0 a	4.6 b	59.7 ab	2.1 ab	126.4	70.3 a	60.5
P-BK	123.0 ab	4.4 b	53.8 ab	1.4 cd	118.2	40.5 b	61.5
P-BU	137.6 a	3.8 d	25.0 c	0.9 e	105.0	47.1 b	58.5
T-BO	115.1 ab	3.8 cd	21.1 c	1.1 de	118.8	37.5 b	44.0
T2-BF	106.2 b	4.0 bc	42.4 bc	1.3 de	94.0	43.6 b	52.7
T2-ES	120.5 ab	4.3 bc	63.2 ab	1.1 de	127.0	56.3 ab	42.5
				2010			
GP217	111.2 a	5.0 a	34.4 b	2.7	128.7	33.3 bc	51.4
HB-1	109.8 a	4.6 ab	67.7 a	1.9	118.8	32.3 bc	59.1
HB-4	99.6 b	4.6 ab	74.0 a	1.6	133.0	29.9 c	49.5
NII-1	100.2 b	4.4 b	84.4 a	1.6	137.2	27.2 c	36.3
NIII-2	96.6 b	4.3 b	79.8 a	1.7	116.5	39.8 bc	54.3
P-BK	94.7 b	4.2 b	72.9 a	1.7	144.6	51.4 ab	53.8
P-BU	92.6 b	4.3 b	63.5 a	1.7	139.5	59.1 a	63.9
T-BO	109.2 a	4.2 b	79.8 a	1.6	130.4	32.5 bc	53.2
T2-BF	107.8 a	4.6 ab	81.3 a	2.3	112.6	48.7 ab	52.1
T2-ES	96.4 b	4.4 b	85.2 a	2.2	119.6	37.3 bc	46.7

Means are calculated from four replicates of 25–35 (in 2009) or about 25 (in 2010) cuttings each. Different letters indicate significant differences between treatments (*p* < 0.05).

**Table 3:** Rooting of pear rootstock stem cuttings in the Orel Region.

Genotype	Cutting Length, mm	Cutting Base Diameter, mm	Rooting, %	Number of Roots >2 mm per Cutting	Mean Length of Roots >2 mm, mm	Cuttings with New Shoots, %	New Shoot Height, mm
				2010			
GP217	120.1 bcd	4.5	31.5 abc	1.9 ab	103.4	50.8 abc	89.0 abc
HB-1	117.7 cd	4.2	15.5 de	1.2 c	91.4	25.8 de	72.3 cd
HB-4	124.7 bc	4.7	36.0 ab	2.1 a	107.9	54.2 abc	108.0 ab
NII-1	119.4 bcd	4.2	19.5 cde	1.4 bc	78.9	30.8 de	68.5 cd
NIII-2	129.7 ab	4.6	44.0 a	1.9 ab	102.1	39.8 cd	85.6 abc
P-BK	111.5 d	4.6	27.5 bc	1.6 abc	111.9	60.0 ab	96.7 abc
P-BU	127.9 abc	4.5	35.5 ab	1.6 abc	105.9	64.8 a	109.7 a
T-BO	121.8 bcd	4.7	24.0 bcd	1.5 abc	108.2	47.9 bc	80.1 bcd
T2-BF	135.8 a	4.5	26.0 bcd	1.8 ab	105.0	63.5 a	92.3 abc
T2-ES	117.5 cd	4.0	11.5 e	1.2 c	80.8	21.7 e	58.0 d
				2011			
GP217	126.5 cde	4.7	23.0 bc	1.9 bcd	-	21.7	57.0 bcd
HB-1	138.8 abc	4.5	26.0 abc	2.4 ab	-	19.2	84.0 a
HB-4	143.3 ab	4.2	32.0 ab	1.6 bcd	-	18.8	53.0 bcde
NII-1	126.0 de	4.2	22.0 bc	1.4 d	-	13.6	71.3 ab
NIII-2	146.3 a	4.9	36.0 a	2.9 a	-	16.7	59.5 bcd
P-BK	117.0 e	4.6	21.0 bc	1.8 bcd	-	33.3	53.3 bcde
P-BU	132.6 bcd	4.7	24.0 abc	2.2 abc	-	29.2	65.6 abc
T-BO	126.7 de	4.4	29.0 ab	1.5 cd	-	27.6	48.9 cde
T2-BF	124.9 de	4.3	17.0 c	1.5 cd	-	11.8	38.5 e
T2-ES	119.4 de	4.4	28.0 abc	1.8 bcd	-	21.4	44.8 de
				2012			
GP217	130.1 ab	4.2	45.0 bc	1.4 cd	-	38.5	6.1
HB-1	138.4 a	4.1	51.0 ab	1.2 d	-	45.2	4.5
HB-4	142.8 a	4.2	65.0 a	1.8 ab	-	58.7	7.5
NII-1	130.7 ab	3.9	34.0 bc	1.2 d	-	28.0	6.0
NIII-2	135.8 a	4.2	31.0 c	2.0 a	-	29.0	5.4
P-BK	131.0 ab	4.1	53.0 ab	1.5 bcd	-	26.0	5.3
P-BU	142.5 a	4.2	43.0 bc	1.7 abc	-	41.5	9.6
T-BO	122.3 b	4.0	49.0 abc	1.4 cd	-	39.8	6.4
T2-BF	132.0 ab	4.5	43.0 bc	2.0 a	-	40.0	6.6
T2-ES	134.4 ab	4.3	54.0 ab	1.4 cd	-	37.1	5.7

Means are calculated from four replicates of 50 (in 2010) or 25 (2011, 2012) cuttings each. Different letters indicate significant differences between treatments (*p* < 0.05).

**Table 4:** GUS activity in transgenic hybrid seedlings of pear rootstock from the harvests of the years 2007–2009 (pmol 4-MU/µg protein/min).

Gene	Line	Harvest of 2009 in the Greenhouse (2010)	Harvest of 2007 in the Field (2010)	Harvest of 2008 in the Field (2011)
*uidA*-intron	HB-1	12.6 ± 3.1		50.1 ± 5.0
		11.6 ± 3.5		100.4 ± 12.0
		10.5 ± 1.5		68.0 ± 4.1
	HA-2	5.1 ± 0.9		
		0.6 ± 0.2		
		1.9 ± 0.5		
	HA-3		10.7 ± 1.3	
			16.8 ± 2.2	
			1.0 ± 0.2	
			13.1 ± 2.6	
	HA-4		6.9 ± 0.6	27.6 ± 5.5
			7.8 ± 1.2	47.4 ± 3.8
			10.7 ± 1.8	41.6 ± 5.0
			8.0 ± 1.0	
	HA-5			12.6 ± 1.1
				29.6 ± 4.4
				11.9 ± 1.5
	HA-6	35.4 ± 5.1	2.2 ± 0.1	
		7.6 ± 1.5	2.2 ± 0.4	
		11.8 ± 2.5	36.0 ± 5.0	
			1.7 ± 0.2	
*uidA*	NII-1	25.7 ± 4.2	1.3 ± 0.2	33.7 ± 7.4
		7.3 ± 1.5	14.9 ± 3.3	41.0 ± 2.1
		10.4 ± 2.5	2.4 ± 0.2	30.5 ± 2.7
			2.0 ± 0.3	
	NII-2	3.9 ± 0.7		
		3.8 ± 0.3		
		7.2 ± 1.7		
	NII-3	5.3 ± 1.1		13.5 ± 1.6
		3.4 ± 0.8		30.6 ± 6.1
		2.4 ± 0.4		25.0 ± 3.2
	NIII-2	1.6 ± 0.4	6.7 ± 1.1	
		1.3 ± 0.3	5.8 ± 1.6	
		4.7 ± 0.9	1.4 ± 0.3	
			5.5 ± 1.0	
	NIII-4	1.7 ± 0.4	5.9 ± 0.6	
		2.0 ± 0.6	1.4 ± 0.4	
		11.2 ± 2.1	21.2 ± 4.0	
			9.6 ± 1.1	
	NIII-5		0.7 ± 0.1	1.1 ± 0.2
			7.9 ± 1.2	19.4 ± 2.1
			2.7 ± 0.5	1.3 ± 0.2
			0.8 ± 0.1	
	NIV-2	19.2 ± 4.0	1.7 ± 0.2	
		4.4 ± 0.8	2.5 ± 0.2	
		1.1 ± 0.2	1.3 ± 0.2	
			3.2 ± 0.5	

Data are expressed as mean ± SD (N = 3).

**Table 5:** GUS activity in transgenic vegetative progeny of pear rootstock (pmol 4-MU/µg protein/min).

Line	Graftings from 2008 in the Field (2009)	Cuttings from 2009 in the Field	
		2010	2011
HB-1	14.3	15.3	14.5
	16.8	17.3	25.1
	20.1	13.6	46.6
HB-4	8.5	2.5	5.9
	5.8	6.1	8.5
	4.3	6.1	11.0
NII-1	20.1	7.9	17.2
	15.3	11.2	25.8
	14.5	12.6	12.8
NIII-2	8.8	3.5	11.0
	7.6	3.1	12.4
	5.7	3.3	15.1
